# Knowledge of obstetric danger signs and associated factors: a study among mothers in Shashamane town, Oromia region, Ethiopia

**DOI:** 10.1186/s12978-020-0853-z

**Published:** 2020-01-16

**Authors:** Biresaw Wassihun, Berhanu Negese, Hunduman Bedada, Solomon Bekele, Agegnehu Bante, Tomas Yeheyis, Agere Abebe, Duro uli, Merima Mohammed, Salasebish Gashawbez, Emebet Hussen

**Affiliations:** 1 Colleges of Medicine and Health Sciences, Arba Minch University, Arba Minch, Ethiopia; 2 Ethiopian Midwifery Association, Addis Ababa, Ethiopia

**Keywords:** Knowledge, Obstetric, Danger sign, Ethiopia

## Abstract

**Background:**

Child birth which is a special moment for parents, families and communities is also a time of intense vulnerability. In many developing countries including Ethiopia, maternal morbidity and mortality still pose a substantial burden. Raising awareness of women about the danger signs of pregnancy and childbirth is the first essential step in appropriate and timely obstetric care.

**Objective:**

To assess the knowledge of obstetric danger signs among mothers and associated factors in Shashamane town, oromia region, Ethiopia.

**Methods:**

A community based cross sectional study design was employed. All kebeles were included in study; the number of households was determined using proportionate-to-population size then systematic random sampling technique to select 422 women who gave birth in Shashamane town between April and May 2018. A structured questionnaire was used to collect data. Data was checked and entered into Epi data version 3.1 then exported to Statistical Package for Social Science version 23 for analysis. Univariate, bivariate and multivariable analysis with 95% CI was carried out. Women who spontaneously mentioned at least two danger signs of pregnancy from eight items were considered to have good knowledge of the obstetric danger signs.

**Result:**

A total of 422 mothers were involved in the study. The mean age of the respondent was 25 with a standard deviation of 4.3 year. 59.5% of the respondents were found to have poor knowledge of obstetric danger signs. Majority of respondents mentioned vaginal bleeding (64.7%) as a danger sign of pregnancy. According to the result of the multivariable analysis, antenatal care was significantly associated with the knowledge of obstetric danger sign. Respondents who attended antenatal care were 1.26 times more likely to have good knowledge of obstetric danger signs than those who had no antenatal care [AOR = 1.26, 95%CI (1.08–1.85)]. Respondents who gave birth at health center were 3.57 time more likely to have good knowledge of obstetric danger signs than those who gave birth at home [AOR = 3.57, 95%CI (1.23–10.39)].

**Conclusion:**

According to this study, the knowledge of obstetric danger signs was poor. Some of the factors associated with this knowledge were antenatal care attendance and place of delivery; therefore, it is recommended that mothers should have at least four antenatal visits; this may create good relationship with the providers and enhance their knowledge. In addition to this providing compassionate and respectful maternity care in health facility is also crucial steps to attract more women to health facilities, and to reduce home deliveries.

## Plain English summary

Worldwide, a projected half million women die as a result of pregnancy and childbirth related complications. In Ethiopia, only one-third of mothers gives birth at a health institution and the maternal mortality ratio is 410/100,000 live births. One of the contributing factors to maternal deaths was a lack of knowledge of pregnancy-related danger signs. Therefore, this study aimed to investigate the knowledge of obstetric danger signs among recently-delivered women and associated factors in Shashamane town, oromia region, Ethiopia. A community based cross-sectional study design was employed on 422 mothers who gave birth in Shashamane town between April and May 2018. The data was collected using a face to face interview and questionnaire. A total of 422 women responded fully for the questions asked. The result of the response was analyzed using statistical software to identify which variables were associated with knowledge of obstetric danger signs. Women attending antenatal care, and giving birth in health facility had better knowledge of obstetric danger sign.

## Background

The burden of pregnancy complications is higher in developing countries [1]. In 2015, a total of 303,000 women have lost their life due to easily preventable pregnancy and childbirth related complications, 99% of which were contributed by low income countries [2]. Knowledge of danger signs during pregnancy, labour and delivery is crucial for safe motherhood [3]. Pregnancy danger signs are those symptoms that may signal danger to a pregnant woman or her fetus and therefore require immediate medical attention. The most common danger signs during pregnancy are severe vaginal bleeding, swollen face/hand, and blurred vision: danger signs during labor and childbirth include severe vaginal bleeding, prolonged labor, and convulsions; Danger signs during the postpartum period include severe bleeding following childbirth, loss of consciousness after childbirth, and fever [4]. Many of the complications that result in maternal deaths contributing to prenatal deaths are unpredictable, and their onset can be both sudden and severe [5, 6]. In low income countries maternal mortality due to childbirth related complication could be prevented if pregnant women recognize danger signs and seek immediate obstetric care [7]. Poor knowledge of danger sign is one of the most common causes of failure to recognize the complication when it occurs and delaying the decision to seek care [8]. The national reproductive strategy of Ethiopia has emphasis on maternal and newborn health to reduce high maternal and neonatal death [8]. The strategy focuses on the need to empower women, men, families and communities to recognize pregnancy related risks, and to take responsibility for developing and implementing appropriate response to them [9]. In many low resources countries, home delivery is prevalent. Therefore, it is important that people are trained to recognize danger signs and develop plans for emergencies [9]. Maternal deaths during child birth has profoundly bad consequences for her family, particularly for children left without care taker and have a negative impact on the society and economies of their nations at large [10]. Majority of maternal deaths are avoidable, if women with complications are able to identify and seek appropriate emergency obstetric care [9]. Maternal deaths have both direct and indirect causes. Around 85% of maternal deaths worldwide are due to direct obstetric complications such as severe postpartum bleeding, infections after delivery, unsafe induced abortion, hypertensive disorders in pregnancy and obstructed labour [11]. Direct cause of maternal mortality was easily avoidable if the mother and family recognize sign and seeking immediate care, health facility provides quality care and transportation were easily available [12]. High levels of maternal mortality can be reduced by providing quality maternity service and empowering women with knowledge of the danger signs of pregnancy and promote appropriate health seeking perceptions. In Ethiopia; however, few studies have been conducted with regarding the knowledge of obstetric danger sign during pregnancy, childbirth and associated factors and we could not find any published research that is conducted in this study area. This study was therefore, aimed at assessing the current status of knowledge of obstetric danger signs and associated factors among mothers in Shashamane town, oromia region, Ethiopia.

## Methods

### Study area

The study was conducted in Shashamane town, located in central part of Oromia region, 130 km from Adama, the capital city of the region and 248 km from Addis Ababa, the capital city of Ethiopia. The town was established in 1903 e. c. Based on the 2007 Census the town has a total population of 218,335 where, about 48.9% are females. It consists of peoples with different languages, more than 18 ethnic groups and foreigners like the Ras Teferian community. There are 74 health institutions in the town. Among this there are two public hospitals which serve the district and referral levels 30 pharmacies six health centers, 28 private clinics, one private hospital and five health posts.

### Study design and period

A community based cross sectional study was conducted in Shashamane town between April and May 2018.

### Population

#### Source population

All mothers who gave birth in the last 1 year at Shashamane town.

#### Study population

All mothers who gave birth within the last 1 year from selected four kebeles of Shashamane town and fulfill inclusion criteria.

#### Inclusion criteria

All mothers who gave birth in the last year in Shashamane town.

Women who were resident of the area for the past 6 months.

#### Exclusion criteria

Mothers who were critically ill and unable to communicate at the time of data collection.

### Sample size determination

The single population proportion formula was used to calculate the sample size by hypothesizing that the proportion of mothers who had good knowledge of danger sign would be 50%, adding a non-response rate of 10%, and using the assumptions of a 95% confidence level and a 5% margin of error. The resulting sample size was 422 mothers.

### Sampling procedure

Systematic random sampling was used to select study participant from 10 kebeles of Shashamane town administration. All kebeles were included in the study and the number of households was determined using proportionate-to-population size.

To select the first household the data collectors used the kebeles administration office and church as a starting point. The data collectors used spinning techniques to select the first house hold by rotating a pen and select the house which is found to the direction of the pen. Then went to the right direction of the first household. From this onwards data was collected in every 12th interval until the desired sample was achieved in each kebeles.

A mother in the selected household was interviewed. For household with more than one individual, only one person was selected using a lottery. For those selected household which was unavailable during data collection, data collectors revisited three times at different time intervals, and if interviewers still failed to get an individual in the house hold, it was considered as non-response.

### Data collection tool and procedure

Data were collected by face to face interview using a structured questionnaire adapted from the survey tools developed by JHPIEGO’s Maternal Neonatal Health Program. The questionnaire was used to assess the knowledge of mothers regarding the pregnancy danger signs. The data collection tool was pre-tested on women with similar characteristics living out of the study area on 10% of sample size. After pre-testing further adjustments to the data collection tool were made to improve clarity. All of the questionnaires were checked for completeness and accuracy before, during and after the period of data collection. Five diploma midwives who were fluent in the local language were involved in the data collection. Two Bachelor of Science degree (BSc) holder health professionals were recruited as supervisors.

### Data quality control

After pre-testing the questionnaire, Chronbach Alpha was calculated by using SPSS window version 23.0 to test internal consistency (reliability) of the item and Chronbach Alpha greater than 0.7 was considered as reliable. On the top of this, content validity was cross checked by another maternal and reproductive health expert at Arba Minch University. Data collectors and supervisors were trained for 2 days on the study instrument and data collection procedure. The principal investigator and the supervisors checked the data for completeness and corrective measures was taken accordingly.

### Data processing and analysis

The collected data were checked manually for completion and any incomplete or misfiled questions, cleaned and stored for consistency, entered into EpiData version 3.1 (EpiData Association, Odense, Denmark), and then exported to SPSS version 23.0 (IBM Corp., Armonk, NY, USA) for analysis. Descriptive statistics were calculated and presented using tables and figures. Multivariable logistic regression analysis was performed to adjust for possible confounding variables. Variables that were significant in the bivariate logistic regression were entered into the multiple regression analysis. The *p* < 0.05 or 95% confidence intervals (CIs) not including 1.0 were considered to indicate statistical significance.

### Measurement

Danger signs are indications of potential obstetric complications. Knowledge of women about obstetric danger signs were measured by the total number of correct spontaneous answers to eight items on knowledge of pregnancy danger signs and eight items on knowledge of labor and childbirth danger signs with a minimum score of zero and maximum of eight. Spontaneous knowledge refers to the respondent’s naming a sign without being asked about that sign by name. Only true obstetric complications spontaneously mentioned by individual respondents were included. Accordingly, two categories were developed for each pregnancy and childbirth danger signs. Good knowledge about pregnancy danger signs: women who spontaneously mentioned at least two danger signs of pregnancy. Poor knowledge about pregnancy danger signs: women who did not spontaneously mention two danger signs of pregnancy. Good knowledge about the danger signs of labor and childbirth: women who spontaneously mentioned at least two danger signs of labor and childbirth. Poor knowledge about the danger signs of labor and delivery: women who did not spontaneously mention two danger signs of labor and childbirth.

## Result

### Socio demographic characteristics

A total of 422 mothers were involved in the study. The mean age of respondents was 25 with a standard deviation of 4.3 years. The minimum and maximum age of respondent was 16 and 39 respectively. Around 154 (36.5%) of the participants were Muslim and 266 (63%) were Oromo in ethnicity. Majority 394 (93.4%) of the women were married and majority 275(65.2%) of the respondents were housewives. 162 (38.4%) had completed primary school and 57(13.5%) had completed college or greater. Majority 243 (57.6%) of the respondents had a monthly family income < 4000 Ethiopian birr (Table 1).

**Table 1:** Socio demographic characteristics of mother in Shashamane town, April, 2018

Variable	Frequency(*n* = 422)	Percent (%)
Age in year		
16–19	19	4.5
20–24	164	38.9
25–29	163	38.8
30 and above	76	18.0
Marital status		
Single	16	3.8
Married	394	93.4
Divorced	11	2.6
Widowed	1	0.2
Ethnicity		
Oromo	266	63
Amara	62	14.5
Wolayita	39	9.2
Sidama	16	3.8
Tigre	13	3.1
Other^a^	27	6.4
Religion		
Muslim	154	36.5
Orthodox	139	32.9
Protestant	124	29.4
Catholic	5	1.2
Occupation		
Unemployed	275	65.2
Employer	43	10.2
Merchant	60	14.2
Private employer	26	6.2
Farmer	1	0.2
Others^b^	17	4
Educational status		
No formal education	69	16.4
Primary school	162	38.4
Secondary and preparatory	134	31.8
Collage and above	57	13.5
Monthly income		
Below 4000	243	57.6
Above 4000	179	42.4
Median 4000		

In ethnicity ^a^ = Guraga, Gamo, Gofa. In occupation ^b^ = students, Daly workers

### Obstetric history related characteristics

Out of 422 respondents, 244 (57.8%) were multi para while other 164 (38.9%) were grand multi para. Majority of the respondents, 368 (87.2%) gave birth at a health center. From the total respondents, 334 (79.5%) had a spontaneous vaginal delivery, while 86 (20.4%) had an instrumental delivery. Almost all 323 (76.5%) of the respondents decided where they gave birth by themselves and 86 (20.4%) respondents had the decision made by their husband, only 13 (3.1%) were decided by their relatives (Table 2).

**Table 2:** Obstetric characteristics of mothers in Shashamane town, April, 2018

Variables	Frequency (*n* = 422)	Percent (%)
Age of first pregnancy in year		
< 20	134	31.8
20–24	275	65.2
25–29	12	2.8
30–34	1	0.2
Total number of pregnancy		
1	178	42.2
2–5	209	49.5
> 6	35	8.9
Pregnancy resulted alive		
0	18	4.3
1	193	45.7
> 1	211	50
Total number of children		
1	14	3.3
2–4	376	89.1
> 5	32	7.6
ANC follow up		
Yes	367	87.0
No	55	13.0
Number of ANC follow up		
One	5	1.3
Two	22	5.9
Three	107	29.1
Four and above	233	63.4

Key: *ANC* Antenatal care

### Knowledge about obstetric danger signs

**Fig. 1:**
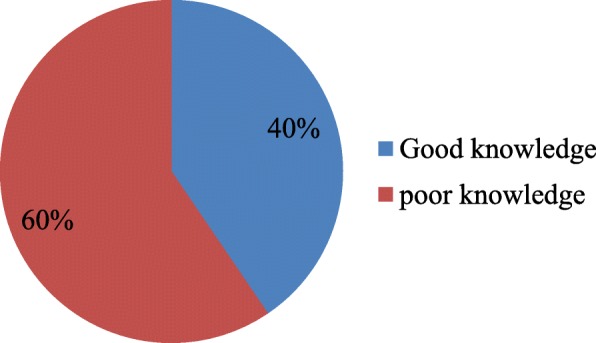
Knowledge of obstetric danger sign among mothers in Shashamane town, April, 2018

Knowledge of obstetric complications was assessed by questions of danger signs related to pregnancy and childbirth. The most commonly mentioned danger sign of pregnancy was vaginal bleeding (64.7%), followed by absent or decreased fetal movements (38.6%). Similarly, the most commonly mentioned danger sign during childbirth was bleeding (60%), followed by absent or decrease fetal movements (28.4%). In addition, a commonly mentioned danger sign during post-partum was postnatal bleeding (63.3%), followed by postnatal fever. 251(59.5%) of the respondents were found to have poor knowledge; however, 171(40.5%) had good knowledge of obstetric danger sign (Table 3), (Fig. 1).

**Table 3:** knowledge of obstetric danger signs during pregnancy, child birth and postnatal among mothers in Shashamane town, April 2018

Variables	Frequency (*n* = 422)	Percent (%)
Danger sign during pregnancy		
Vaginal bleeding		
Yes	273	64.7
No	149	35.3
Persistent nausea and vomiting		
Yes	121	28.9
No	301	71.1
Swelling of body		
Yes	124	29.4
No	298	70.6
Persistent headache and blurred vision		
Yes	164	38.9
No	258	61.1
Absent or decreased fetal movement		
Yes	163	38.6
No	259	61.4
Severe abdominal cramps		
Yes	121	28.9
No	301	71.1
Leakage of amniotic fluid without lab		
Yes	119	28.1
No	303	71.9
High fever		
Yes	98	23.2
No	324	76.8
Danger sign during child birth		
Bleeding through birth canal		
Yes	253	60
No	169	40
Persistent headache &blurred vision		
Yes	105	24.9
No	317	75.1
Swelling of hand, face etc.		
Yes	54	12.8
No	368	87.2
Absent or decrease fetal movement		
Yes	120	28.4
No	302	71.6
Increased blood pressure		
Yes	74	17.5
No	348	82.5
Retained Placenta (> 1 h.)		
Yes	82	19.4
No	340	80.6
Mal-presentation/position		
Yes	54	12.8
No	368	87.2
Severe continuous abdominal pain		
Yes	120	28.4
No	302	71.6
Danger sign during post-partum		
Post Natal Bleeding		
Yes	267	63.3
No	155	36.7
Persistent headache &blurred vision		
Yes	106	25.1
No	316	74.9
Swelling hand, face		
Yes	99	23.5
No	325	76.5
Postnatal Fever		
Yes	168	38.9
No	254	60.2
Unconscious		
Yes	90	21.3
No	332	78.7

### Factors affecting knowledge of obstetric danger signs

According to the bivariate analysis, knowledge was significantly associated with educational status of mothers. Respondents who had a formal education were 6.01 times more likely to have good knowledge of obstetric danger signs than respondents did not have formal education [COR = 6.01 (95% CI 2.26–2.60)]. Place of delivery was significantly associated with knowledge of obstetrics danger signs, respondents who gave birth at a health institution were 5.7 times more likely to have good knowledge of obstetric danger signs than those who gave birth at home [COR = 5.7 (95%CI 2.98–11.17)]. Occupation of respondent was also significantly associated with knowledge of obstetric danger signs. Respondents who were working for a governmental employer were 1.36 times more likely to have good knowledge of obstetric danger signs than housewives [COR = 1.36 (95%CI 1.853–2.17)]. In multivariate logistic regression on both socio-demographic and obstetric history of respondents, number of antenatal care (ANC) visits and place of delivery were found to be significantly associated with knowledge of obstetric danger signs at *P*-value of < 0.05. Respondents who attended ANC were 1.26 times more likely to have good knowledge of obstetric danger signs than respondent with no ANC [AOR = 1.26 (95%CI 1.07–2.85)]. Respondents who gave birth in the health center had 3.57 time more likely to have good knowledge of obstetric danger signs than those respondents who gave birth at home [AOR = 3.57 (95%CI1.23–10.39)] (Table 4).

**Table 4:** Factors associated with knowledge of key obstetric danger sign during pregnancy, child birth and postnatal period among mothers in Shashamane town, April 2018

Types of variable	Good knowledge	Poor knowledge	COR (95% C.I)	AOR (95% C.I)
Age of respondent				
15–19	10	9	1	1
20–24	56	108	1.11 (0.41–3.04)	1.46 (0.24–8.98)
25–29	67	96	0.52 (0.29–0.90)	0.84 (0.26–2.67)
≥ 30	38	38	0.69 (0.04–1.21)	1.28 (0.46–3.51)
Occupation				
House wife	118	157	1	1
Governmental employed	18	27	1.36 (1.85–2.17)^a^	2.05 (0.97–4.32)
Self-employed	37	67	1.07 (0.51–2.24)	1.74 (0.55–5.51)
Number of pregnancy				
1	70	108	1	1
2–5	83	126	0.61 (0.29–1.27)	1.77 (0.37–6.68)
≥ 6	18	17	0.62 (0.30–1.27)	0.98 (0.27–3.62)
ANC follow up				
No	51	4	1	1
Yes	120	247	0.67 (0.45–1.54)	1.26 (1.07–1.85)^a^
Place of delivery				
Home	41	13	1	
Health center	130	238	5.74 (2.98_11.16)^a^	3.57 (1.23–10.39)^a^
Source of information				
Health personal	32	204	1	1
Relative	5	8	0.03 (0.02–0.06)	0.03 (0.02–0.06)
Friends	2	10	0.14 (0.04–0.45)	0.28 (0.05–0.17)
Media	123	29	0.04 (0.09–0.21)	0.05 (0.01–0.31)
Educational status				
Informal education	16	7	1	1
Formal education	126	167	6.07 (2.26–16.26)^a^	3.91 (0.84–18.12)
Collage and above	29	77	2.03 (1.23–3.25)^a^	1.96 (0.95–4.05)
Income				
< 4000	106	135	1	1
> 4000	65	114	1.35 (1.91–2.02)^a^	1.11 (0.60–2.06)

## Discussion

The findings of this study revealed that the good knowledge of respondents regarding obstetric danger sign was 40% which is higher than other studies conducted in Egypt 26.0% [12], Jordan 15.2% [13], and Uganda 19% [14]. However, it was lower than the findings of South Africa 52% [15]. This difference might be due to socio-cultural differences and differences in implementation of health programs. The finding of this study is also consistent with study conducted in another part of the Ethiopia in Debra Berhan public health institution, 38.6% [16].

The most common danger sign mentioned during pregnancy was vaginal bleeding at 64.7%. This is greater than in Tsegedie district, Tigray 51.2% [17]. The difference may be due to study period difference and increasing the number of staffs which providing counseling on danger signs during antenatal, childbirth and postnatal period. The finding of this study was comparable with the same study conducted in Debre berhan city administration 61.1% [18]. Vaginal bleeding during childbirth was also mentioned by about 60% of the respondents, while persistent headache and blurred vision was mentioned by 24.9%, which was higher than study conducted in aleta wendo district, sidama zone 55 and 7%. The discrepancy may be due to improvement of service provision in maternity care [19]. In this study respondents who had a history of ANC were 1.26 times more knowledgeable than those who had no ANC visits. This finding is consistent with study done in other part of Ethiopia [17, 19]. Simlarly in this study respondents who gave birth in health faclity was 3.57 times more likely to have good knowledge than those who gave birth at home.

## Conclusions

This study’s findings revealed that knowledge about obstetric danger signs of pregnancy overall was poor. The most commonly mentioned danger signs during pregnancy, and childbirth was severe vaginal bleeding followed by absence of fetal movements. From this study it can be concluded that women’s knowledge of danger signs during pregnancy and childbirth was increased by their educational level, number of ANC visits and having an institutional delivery. We recommended mobilizing communities to increase knowledge on obstetric danger sign during pregnancy. Similarly encouraging pregnant women to attend antenatal clinics and providing health information dissemination related to pregnancy danger sign and seeking behavior is also vital. Finally, we recommend further research to be conducted using both qualitative and quantitative methods to address the behavior and attitude of health care providers and maternity users regarding obstetric danger sign.

## Data Availability

The datasets used and/or analyzed during the current study available from the corresponding author on reasonable request.

## Abbreviations

ANC: Antenatal care
AOR: Adjusted odds ratio
CI: Confidence interval
COR: Crude Odds ratio
